# Supplementary material for: Strong Exciton–Phonon Coupling as a Fingerprint of Magnetic Ordering in van der Waals Layered CrSBr
Source: ACS Nano. 2024 Jan 19;18(4):2898–905. doi: 10.1021/acsnano.3c07236 (PMC10832030; doi:10.1021/acsnano.3c07236)
Supplement: Supplementary file 1 — nn3c07236_si_001.pdf [file nn3c07236_si_001.pdf]

Supporting Information

Strong Exciton-Phonon Coupling as a Fingerprint of Magnetic Ordering in van der Waals Layered CrSBr

Kaiman Lin[1,2], Xiaoxiao Sun[2], Florian Dirnberger[3], Yi Li[2,4], Jiang Qu[5], Peiting Wen[2,4], Zdenek Sofer[6], Aljoscha Söll[6], Stephan Winnerl[2], Manfred Helm[2,4], Shengqiang Zhou[2], Yaping Dan[1,*], Slawomir Prucnal[2,*]

[1] University of Michigan-Shanghai Jiao Tong University Joint Institute, Shanghai Jiao Tong University, 20024 Shanghai, P. R. China

[2] Helmholtz-Zentrum Dresden-Rossendorf, Institute of Ion Beam Physics and Materials Research, Bautzner Landstrasse 400, 01328 Dresden, Germany

[3] Institute of Applied Physics and Würzburg-Dresden Cluster of Excellence ct.qmat, Technische Universität Dresden, Germany

[4] Technische Universität Dresden, 01062 Dresden, Germany

[5] Leibniz Institute for Solid State and Materials Research Dresden (IFW Dresden), Helmholtzstraße 20, 01069 Dresden, Germany

[6] Department of Inorganic Chemistry, University of Chemistry and Technology Prague, Technická 5, 16628 Prague 6, Czech Republic

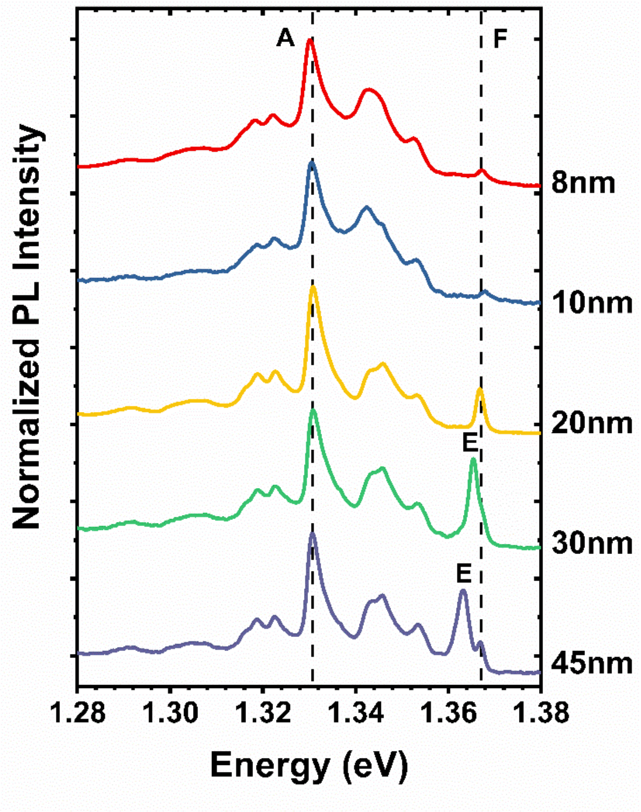

Figure S1. PL spectrum obtained at 4 K for 8nm, 10nm, 20nm, 30nm, and 45nm thick CrSBr

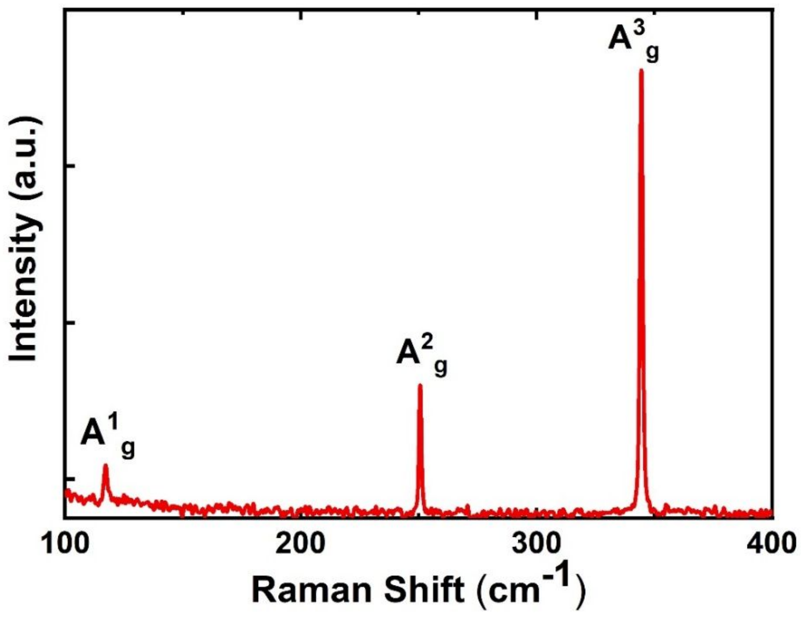

Figure S2. Raman spectrum for 532 nm excitation at 4 K. The Raman spectrum is measured with excitation laser polarized along the b axis, showing the out-of-plane A^1_g mode ~ 118 cm^{-1} (14.6 meV), A^2_g mode ~ 251 cm^{-1} (31.1 meV) and A^3_g modes ~ 345 cm^{-1} (42.8 meV).

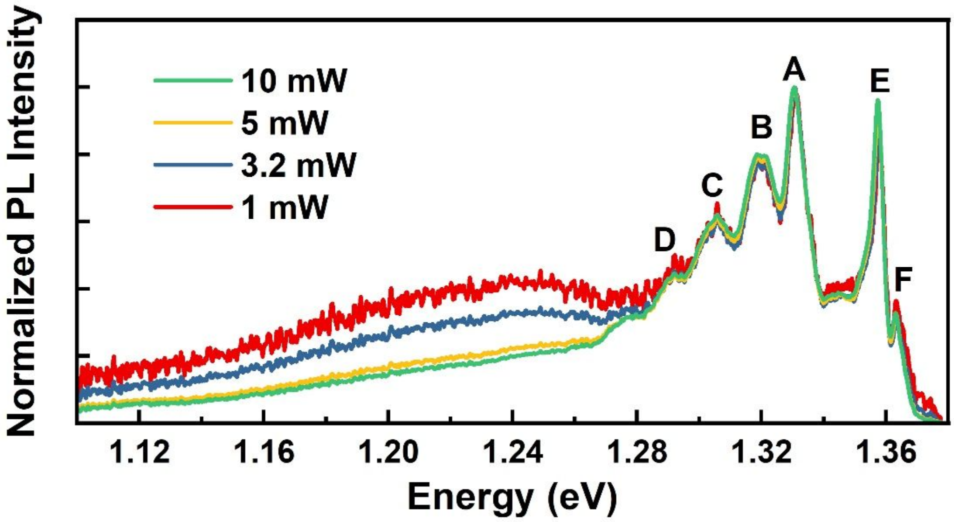

Figure S3. Normalized Power-dependent PL spectra for 532 nm excitation at 4 K. The multi-peaks remain proportionally unchanged with increasing pumping power, indicating that the broad PL spectrum contains and only contains exciton-phonon and exciton-polariton states.

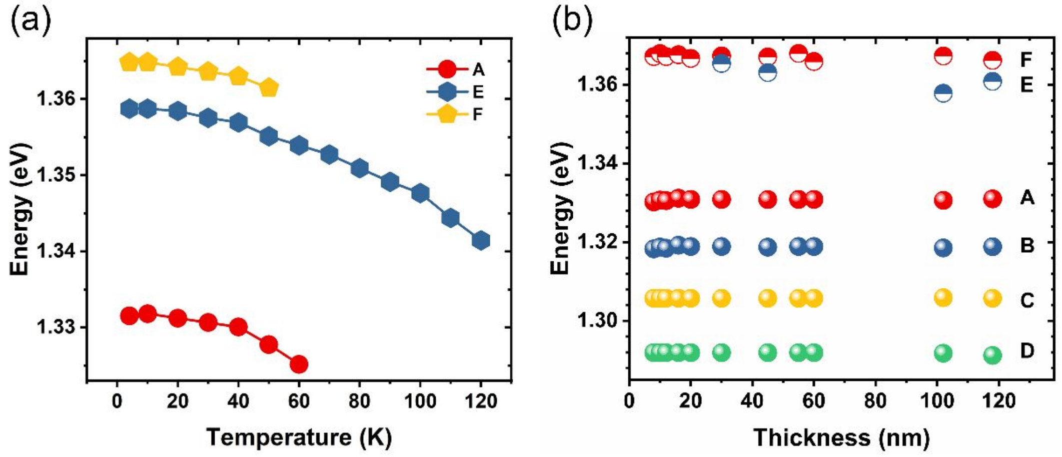

Figure S4. (a) Evolution of peak A, E and F from 4 K to 120 K. **(b)** Energy of peaks in PL spectra obtained at 4 K for CrSBr as a function of thickness. The circles are energies obtained from Lorentzian fitting.

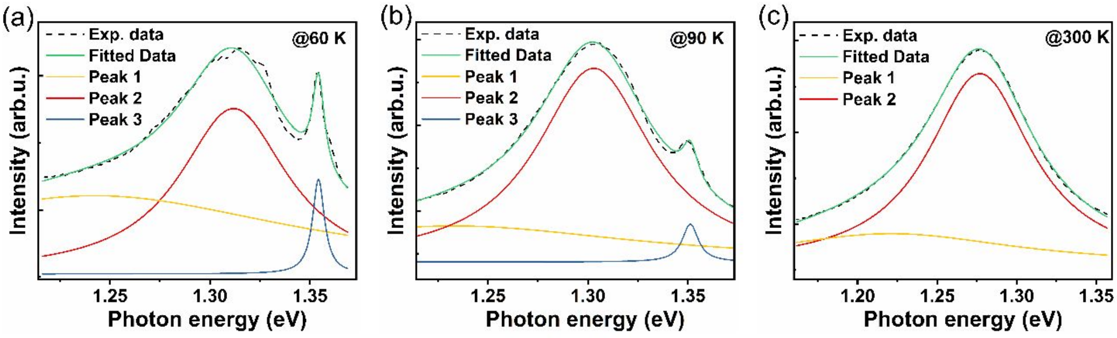

Figure S5. PL spectrum and Lorentzian Fitting curves of CrSBr acquired at **(a)** 60 K **(b)** 90 K and **(c)** 300 K.

The data presented in Figure 3 of the main text were obtained by fitting the experimental PL spectra with Lorentzian functions. It shows the FWHM and peak position of the main emission across the

temperature range of 60 K to 300 K, and integrated PL intensity from 4 K to 300 K. Figure S5 demonstrate this fitting procedure for three representative PL spectra acquired at 60 K, 90 K and 300 K. Notably, the PL data below 60 K were not included in the analysis of peak position and FWHM due to the distinct splitting in different phonon sideband emissions. Nevertheless, the collected data from 60 K to 300 K are adequate for elucidating the exciton-phonon coupling mechanism in the investigated AFM CrSBr, with a Néel temperature (T_N) at around 135 K. For PL spectra taken at 60 K and 70 K, we approximated the data with a broad peak encompassing individual phonon-sideband emissions. Spectra with a distinct peak at approximately 1.35 eV were fitted with three Lorentzian functions: the first for background compensation, the second representing the main emission from exciton-phonon coupling, and the third (identified as E in the main text) linked to exciton-polariton states from strong coupling between excitons and photons in our 102 nm-thick CrSBr crystal. PL spectra above the T_N (above 140K) were fitted using two Lorentzian functions. As for the integrated PL intensity across the temperature range of 4 K to 300 K, it was the integration of the fitted Lorentzian functions representing the exciton-phonon coupling branch at each temperature.

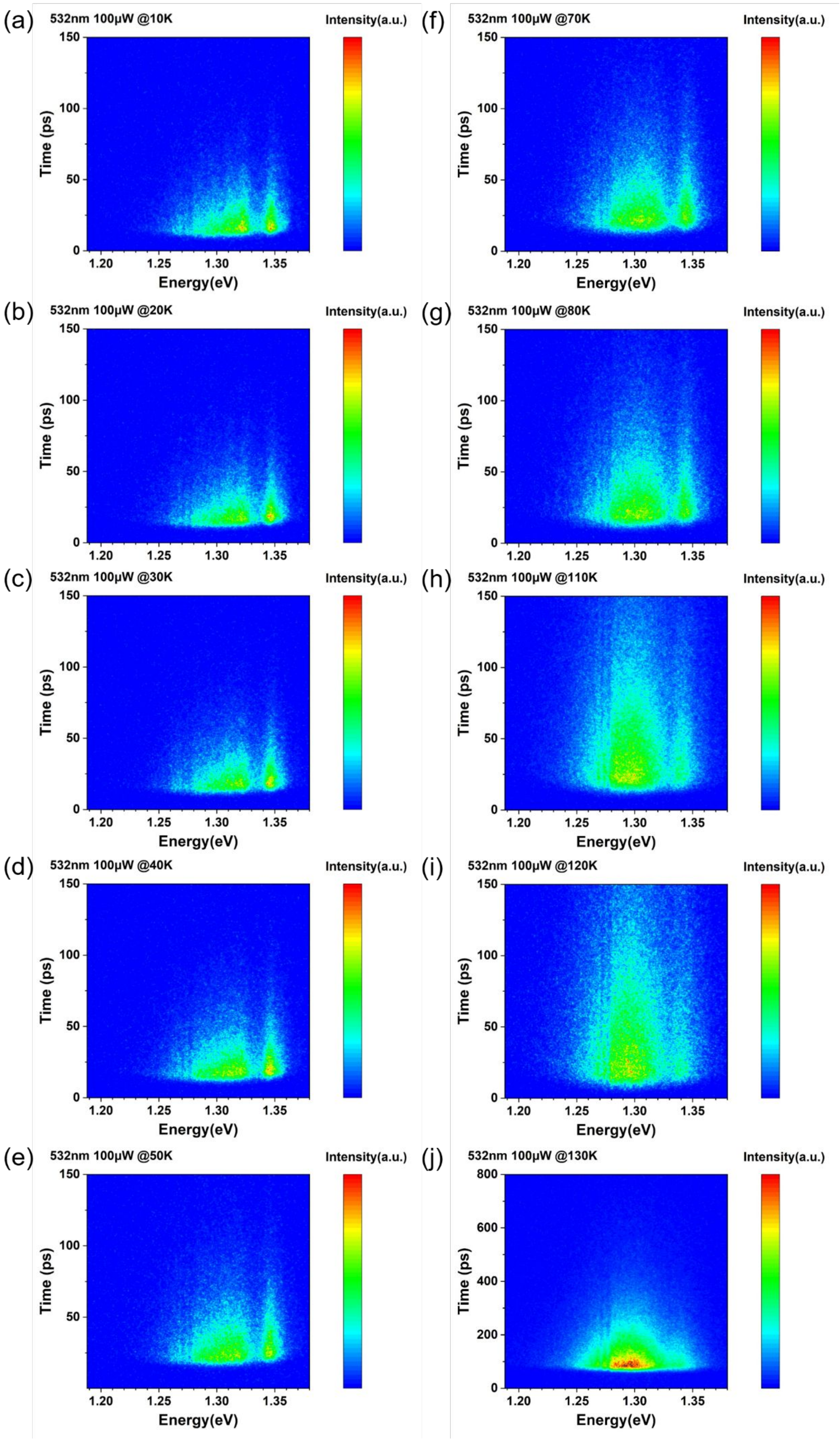
(a)
532nm 100μW @10K
(b)
532nm 100μW @20K
(c)
532nm 100μW @30K
(d)
532nm 100μW @40K
(e)
532nm 100μW @50K
(f)
532nm 100μW @70K
(g)
532nm 100μW @80K
(h)
532nm 100μW @110K
(i)
532nm 100μW @120K
(j)
532nm 100μW @130K
Intensity(a.u.)
Time (ps)
Energy(eV)

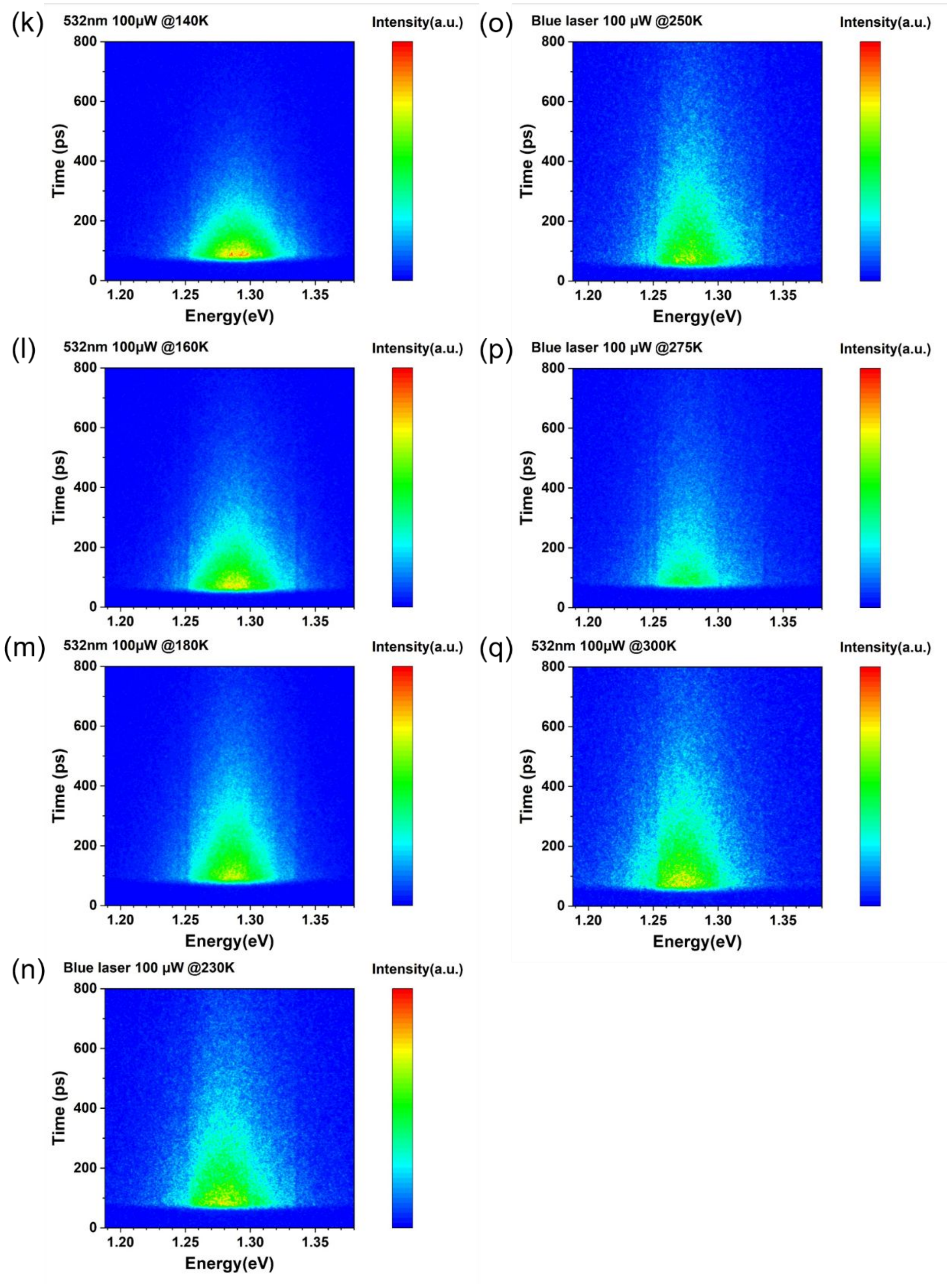

Figure S6. Spectrally-resolved streak-camera image of the PL at temperatures of **(a)** 10 K, **(b)** 20 K, **(c)** 30 K, **(d)** 40 K, **(e)** 50 K, **(f)** 70 K, **(g)** 80 K, **(h)** 110 K, **(i)** 120 K, **(j)** 130 K, **(k)** 140 K, **(l)** 160 K, **(m)** 180 K, **(n)** 230 K, **(o)** 250 K, **(p)** 275 K, **(q)** 300 K.
